# Determination of the Shear Buckling Load of a Large Polymer Composite I-Section Using Strain and Displacement Sensors

**DOI:** 10.3390/s121216024

**Published:** 2012-11-22

**Authors:** Jin Y. Park, Jeong Wan Lee

**Affiliations:** 1 Mechanical Engineering, Minnesota State University, Mankato, MN 56001, USA; E-Mail: jin.park@mnsu.edu; 2 Mechanical and Mechatronics Engineering, Kangwon National University, 1 Kangwondaehak-gil, Chuncheon-si, Gangwon-do 200-701, Korea

**Keywords:** strain sensor, displacement sensor, shear buckling, polymer composite I-section, asymmetric loading

## Abstract

This paper presents a method and procedure of sensing and determining critical shear buckling load and corresponding deformations of a comparably large composite I-section using strain rosettes and displacement sensors. The tested specimen was a pultruded composite beam made of vinyl ester resin, E-glass and carbon fibers. Various coupon tests were performed before the shear buckling test to obtain fundamental material properties of the I-section. In order to sensitively detect shear buckling of the tested I-section, twenty strain rosettes and eight displacement sensors were applied and attached on the web and flange surfaces. An asymmetric four-point bending loading scheme was utilized for the test. The loading scheme resulted a high shear and almost zero moment condition at the center of the web panel. The web shear buckling load was determined after analyzing the obtained test data from strain rosettes and displacement sensors. Finite element analysis was also performed to verify the experimental results and to support the discussed experimental approach.

## 1. Introduction

An I-section beam consists of two panels of flanges and a web panel. When loaded, the I-sections are subjected to combined axial (or normal) and shear stress fields. It is well-known that the web panel does most of work in resisting the shear force [1,2]. The shear force often causes a critical condition of failure of a loaded I-section. For instance, inclined cracks or damage patterns are easily observed from tested metal I-sections. The inclined cracks or patterns in a deep beam show the existence of shear in the web. If a web height is large enough (a deep beam with span/depth < 6), member capacity is usually governed by the shear behavior of the web material.

Since the early 1990s, many researchers have studied the buckling of fiber reinforced polymer (FRP) composite members: Barbero and Raftoyiannis reported analytical and experimental approaches to buckling of pultruded FRP columns [1,2]. Motram [3] published his study on pultruded composites’ lateral-torsional buckling for the first time. Bank [4] and his colleagues published a paper describing their effective lateral buckling test method for composite beams. In 2002, Roberts [5] published that a shear deformation could significantly influence the buckling behavior of a composite I-section under any loading scheme. Shan and Qiao [6] proposed a test method and theoretical verification using energy theory for flexural torsional buckling of an open channel beam. Many other recent papers [7–11] have described theoretical and experimental approaches to buckling of composite members and concluded by mentioning the important effect of shear behavior on buckling. However, a full experimental approach to obtain the shear buckling load of large polymer composite I-sections has not been studied much yet. This is because a pure shear stress state is very difficult to achieve experimentally with a large span composite I-section.

In this study, a test method to detect and determine the critical shear buckling load and to observe the buckling behaviors of a comparably large pultruded composite I-section was discussed. An asymmetric shear loading scheme was proposed and utilized for the test program. In order to sensitively obtain the shear buckling load and shear behavior of the composite I-section, twenty strain rosette sensors were attached on the web surface of the I-section. The tested section was also instrumented at various locations with displacement sensors to indicate lateral and vertical displacements of the web. Finite Element Analysis (FEA) was also performed. The results from the FEA were compared with those from the experimental test to verify and support the discussed experimental method.

## 2. Experimental Section

### 2.1. The Tested I-Section

**Figure 1.**
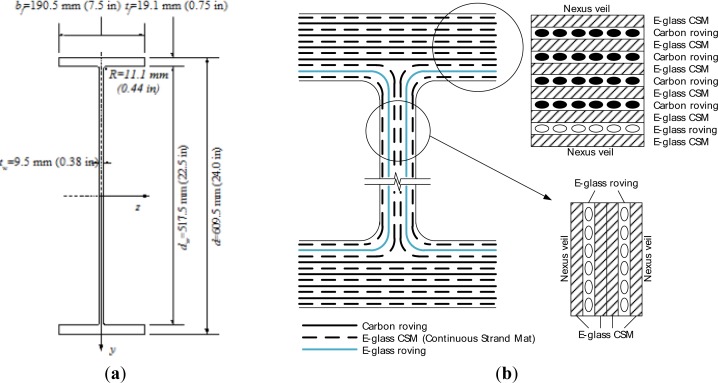
The cross-sectional dimensions and reinforcing scheme of the test component. (**a**) Cross sectional dimension; (**b**) Reinforcing scheme.

The tested composite girder was a 4,572 mm long I-shaped section having the cross-sectional dimensions shown in Figure 1(a). The web was reinforced with a total of six layers: these are two layers of E-glass rovings and three layers of randomly oriented E-glass Continuous Strand Mat (CSM) not 6. The top and bottom flanges were reinforced with eleven E-glass/carbon hybrid rovings combined with E-glass CSM layers for each; four layers of carbon roving, a layer of E-glass roving and six layers of E-glass CSM. The reinforcing scheme for the web and flanges are shown in Figure 1(b).

The weight fractions of the composite constituents of the web were examined following the technique described in Ye *et al.*[12], which was based on ASTM D 2584 [13]. Regarding the weight fractions of the constituents of the flanges, the technique described in ASTM D 3171 [14] was used. Since the carbon fibers are oxidized during a burn-out process, the technique outlined in Ye *et al.*[12] could not be utilized for the flange elements. Thus, digestion in nitric acid 70% solution was performed. After digestion for 168 h (1 week), the carbon and glass fibers were rinsed with acetone and water. The average weight and computed volume fractions of all constituents of the web and the flange elements are listed in Table 1.

### 2.2. Material Properties

**Figure 2.**
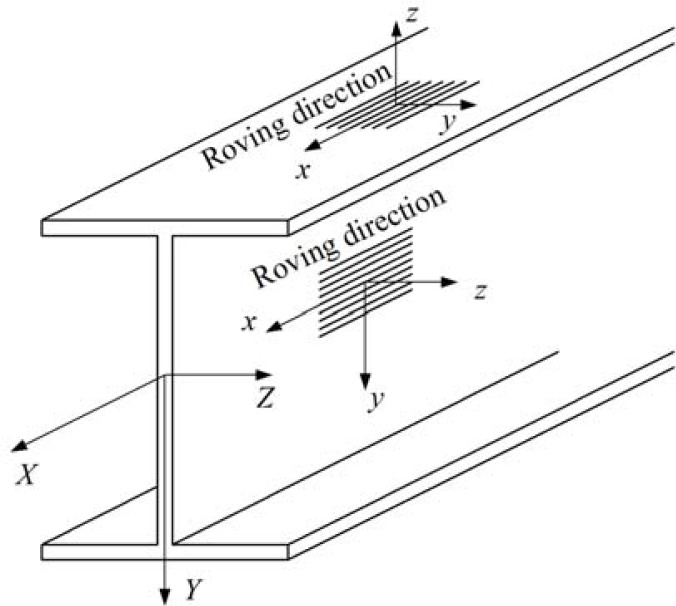
Global and local coordinate system.

Figure 2 shows both the member’s global coordinate system (X, Y, Z) and the member elements’ local coordinate system (*x*, *y* and *z*). The tensile, compressive and shear properties of the web materials of the pultruded composite I-shaped section specimen were determined according to ASTM D 3039 [15], ASTM D 3410 [16] and ASTM D 5379 [17], respectively.

The mechanical properties of the flange material of the specimen were also determined. Since the flanges do not have symmetric lay-ups, a flexural coupon test [18,19] was also carried out to obtain the extensional properties of the flange. For this flexural coupon test, twenty samples were cut from the top and bottom flanges in the *x*-direction. The obtained mechanical properties of the flange material are tabulated in Table 2.

$E_{x}^{b}$, $\sigma_{x}^{ub}$ and $\varepsilon_{x}^{ub}$ in the table are flexural modulus, flexural ultimate strength and strain of the flange in the *x*-direction, respectively. The test results from the flexural tests can be divided into two groups (Group I and Group II) according to the lay-up sequence shown in Table 2.

The material properties of the web material are summarized in Table 3. $E_{x}^{t}$, $\sigma_{x}^{ub}$, $\varepsilon_{x}^{ub}$ and $\nu_{xy}^{t}$ in the table are extensional modulus, ultimate strength, ultimate strain and Poison’s ratio of the web in the *x*-direction, respectively. $E_{x}^{t}$, $\sigma_{x}^{ub}$, $\varepsilon_{x}^{ub}$ and $\nu_{xy}^{t}$ in the table are extensional modulus, ultimate strength, ultimate strain and Poison’s ratio of the web in the *y*-direction, respectively. The superscript “*t*” indicates tensile loading direction. The compressive modulus, compressive ultimate strength, compressive ultimate strain and Poison’s ratio of the web are also listed in Table 3. The superscript “c” indicates compressive loading direction. Shear modulus (G*_xy_*), strength ( $\tau_{xy}^{u}$) and strain ( $\gamma_{xy}^{u}$) are also shown in Table 3.

### 2.3. Experimental Setup

**Figure 3.**
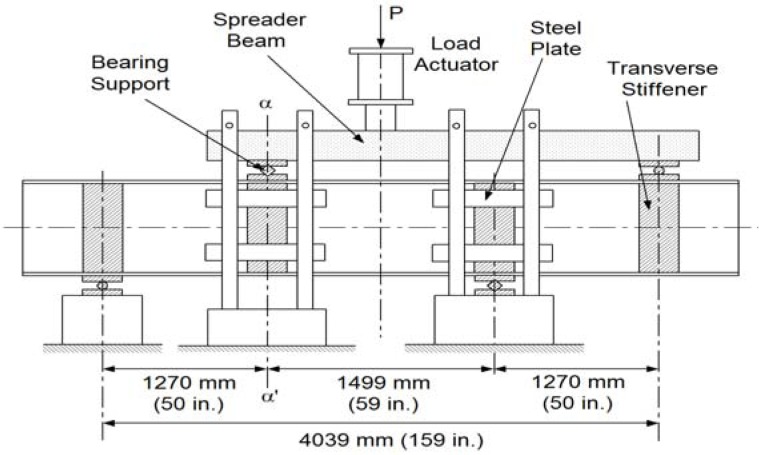
Asymmetric loading test setup.

**Figure 4.**
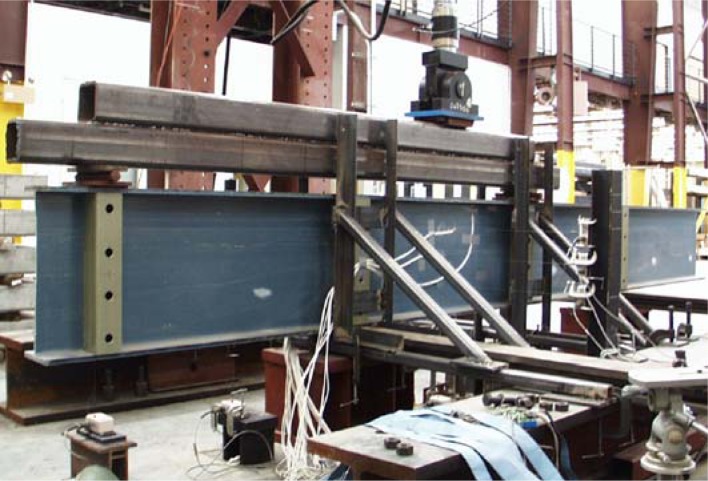
A photograph of asymmetric four point loading setup.

Figure 3 shows a sketch of the test component subjected to four point asymmetric loading. Due to the asymmetric loading pattern, there is the maximum vertical shear force applied at the center of the specimen. Bending moment should be theoretically zero at the center of the beam. At the support and load application points, transverse stiffeners on both sides of the web were provided. A total of eight E-glass/vinylester tubular sections having nominal dimensions of 101.6 mm × 101.6 mm × 9.53 mm were used as transverse stiffeners. The girder was restrained against lateral movement by means of a total of eight steel plates having dimensions of 76.2 mm × 381 mm × 12.7 mm and the transverse stiffeners at the support and load application points. A spreader beam was placed between the load actuator and two bearing plates to apply the asymmetric loading to the specimen. By this loading mechanism, the applied load *P* was divided into 0.73 *P* and 0.27 *P* at the shown bearing contacts. A photograph of the test setup is shown in Figure 4.

### 2.4. Sensor Application

**Figure 5.**
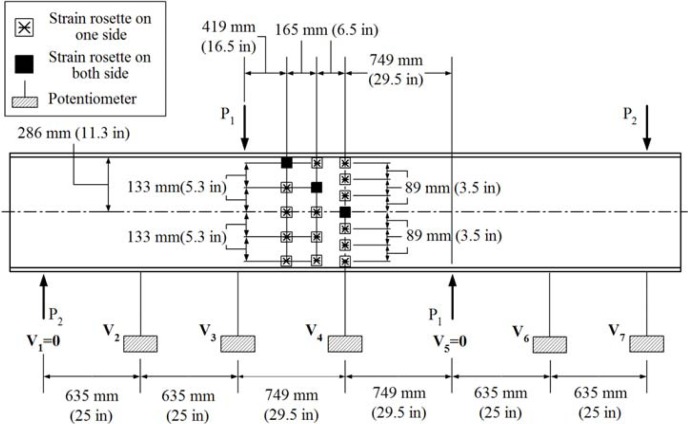
Locations of strain rosettes and displacement sensors on the bottom flange.

**Figure 6.**
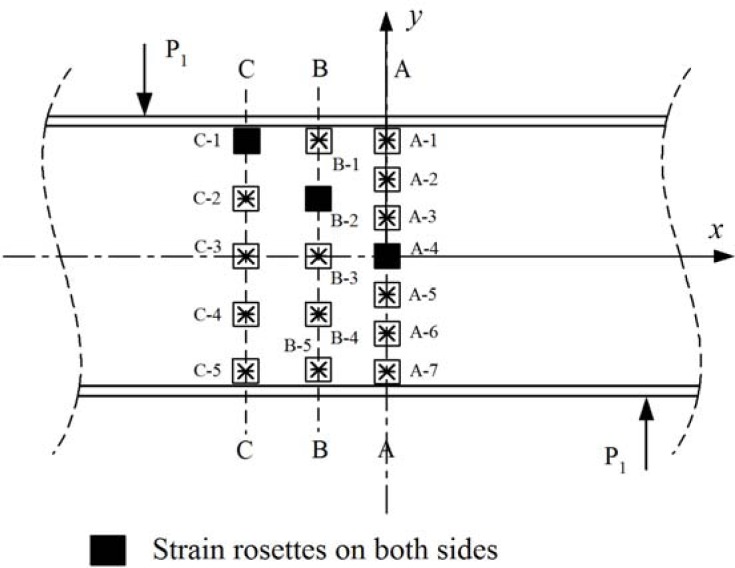
The identification of strain rosettes on the web surface.

**Figure 7.**
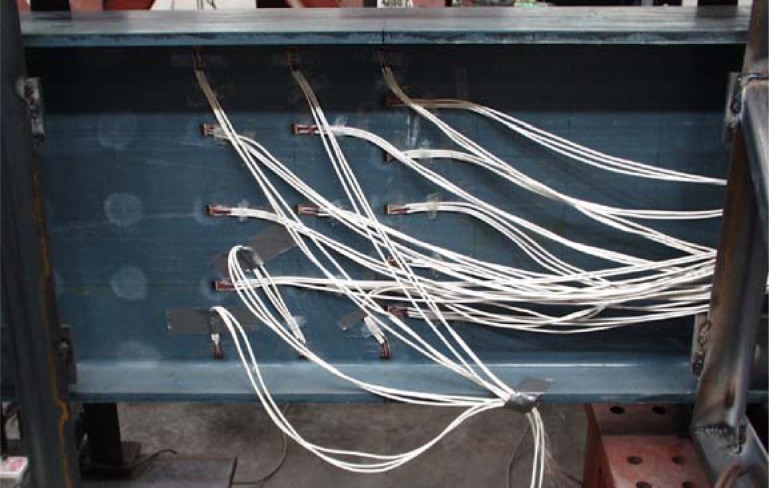
A photograph of strain rosettes on the web.

The instrumentation in the girder consisted of strain rosettes manufactured by Vishay (CEA-125-UR-350) and displacement sensors that were Celesco PT1A string potentiometers with up to 254 mm measurement capacity and accuracy of 0.15% of full stroke range. Twenty strain rosette sensors were used to measure −45°, +45° and 0° directional strains as shown in Figure 5. Seventeen strain rosettes were attached on one side of the web and the other three strain rosettes were located on the other side of the web. The identifications of the strain rosettes are shown in Figure 6. Three locations (A-4, B-2 and C-1) were selected for back-to-back strain rosette applications to accurately determine the strains near buckling. Out-of-plane bending could also be observed by the measurement of the strains from the back-to-back rosettes at these points. Seven strain rosettes were aligned vertically on the center line A-A. Five strain rosettes were located on line B-B 165.1 mm from A-A. Five strain rosettes are also located on line C-C 330.2 mm from A-A. A photograph of the rosettes installed on the web surface is shown in Figure 7.

A total of eight displacement sensors were used to measure the vertical and lateral deflections of the web. Seven locations, including two supporting points, were selected to measure vertical deflections using the potentiometers. They were V_1_, V_2_, V_3_, V_4_, V_5_, V_6_ and V_7_ as shown in Figure 6. The vertical deflections at the supporting points (V_1_ and V_5_) were obviously zero. The locations of the potentiometers are also shown in Figure 7.

**Figure 8.**
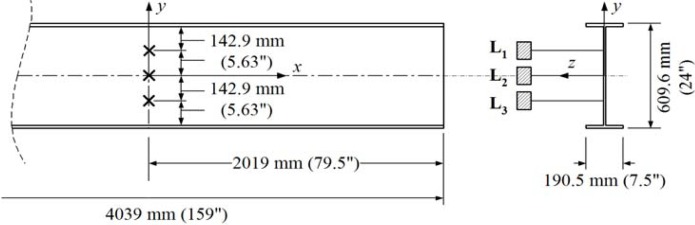
Locations of displacement sensors on the web surfaces.

Figure 8 shows the locations of three additional displacement sensors used to measure the lateral deflections (L_1_, L_2_ and L_3_) of the web at the center of the I-shaped section.The load was applied to the top of the load spreader as shown in Figures 4 and 5. The applied load was continuously increased to the failure point at which the web-flange junction fractured. An MTS 894 kN capacity load generator was used. The girder then was unloaded. The loading and unloading rates were 1.27 mm/s. The data were recorded electronically at a rate of 0.5 Hz.

## 3. Results and Discussion

### 3.1. Test Results

**Figure 9.**
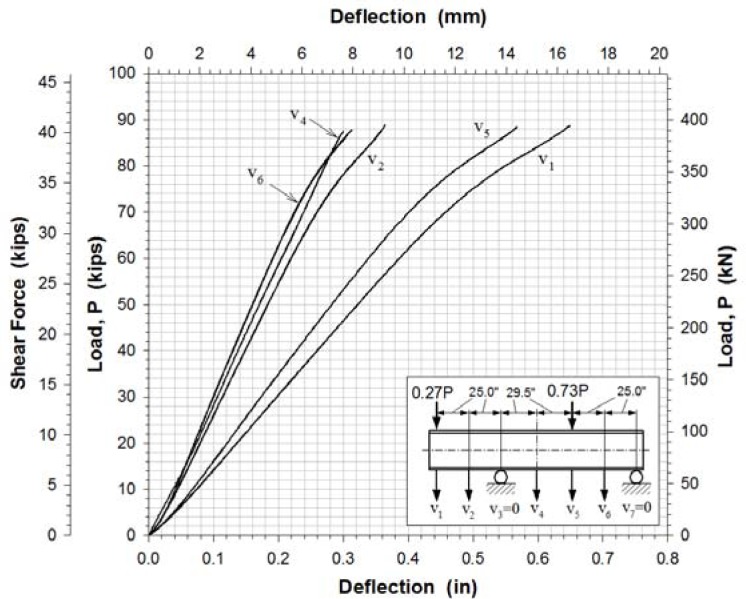
Measured bottom flange vertical displacements.

Load-vertical deflection curves at five locations along the bottom of the lower flange are shown in Figure 9. The vertical deflections were measured using displacement sensors.

**Figure 10.**
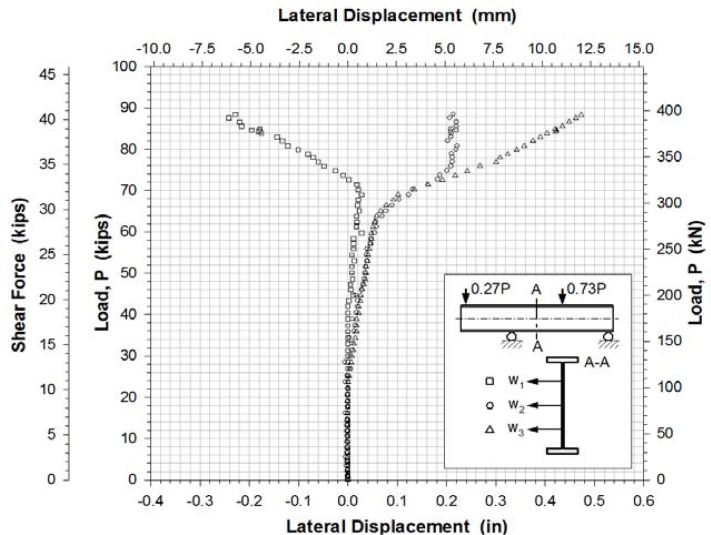
Web out-of-plane displacements at section A-A.

Figure 10 shows the lateral web displacements measured by potentiometers at three locations of the center of the girder.

**Figure 11.**
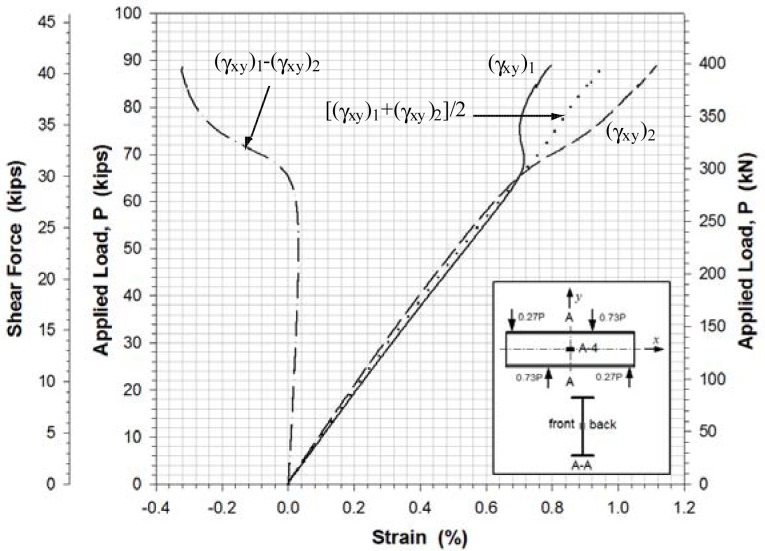
Load-shear strain at location A-4.

**Figure 12.**
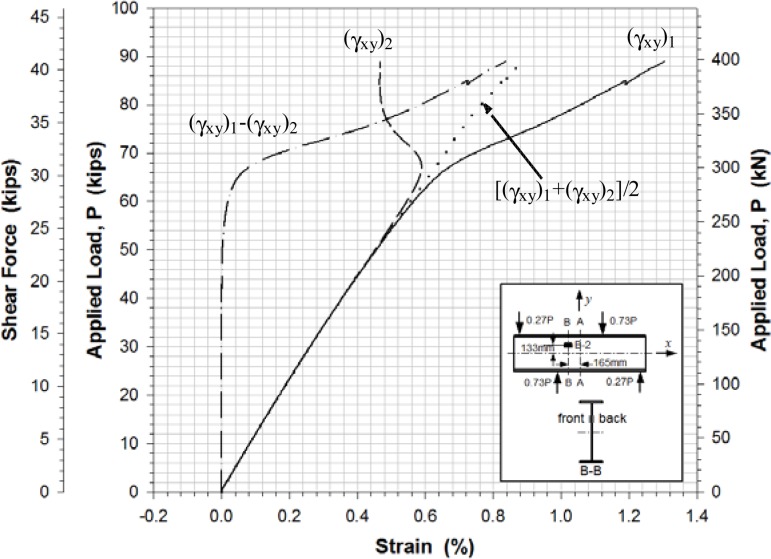
Load-shear strain at location B-2.

**Figure 13.**
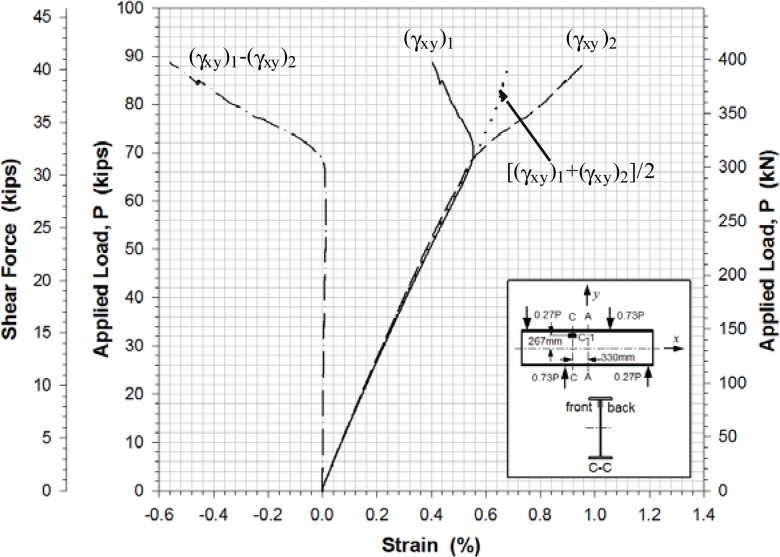
Load-shear strain at location C-1.

Figures 11–13 show the shear strains in the *x*-*y* plane γ*_xy_*, the difference in the shear strain values obtained from the back-to-back gages (γ*_xy_*)_1_ and (γ*_xy_*)_2_, and the average value of the shear strain γ*_xy_* at locations A-4, B-2 and C-1, respectively. All Figures show clearly that prior to buckling the load-strain behavior were close to linear. The values of γ*_xy_* were determined along with the axial strains from Equation (1) and the strain values recorded from the strain rosettes:

(1) $$\varepsilon_{i}=\varepsilon_{x}{\text{cos}}^{2}\theta_{i}+\varepsilon_{y}{\text{sin}}^{2}\theta_{i}+\gamma_{xy}\text{cos}\theta_{i}\text{sin}\theta_{i}(i=1,2,3)$$

where *ε_i_*’s are direct strain sensor readings from three sensors in a rosette, and *θ_i_*’s are the orientation of the three strain gages.

### 3.2. Determination of Shear Buckling Load

The experimental buckling load was estimated from the load-lateral displacement curves obtained from the displacement sensors (Figure 10) and from the load-strain relation curves determined by the data points of strain rosettes (Figures 11–13). It was determined by taking the intersection point of the tangent drawn to a curve before and after buckling as outlined by Hoff, *et al.*[20]. This method is a well-known method called Bifurcation method. It is based on the phenomenon that the web surface should be sharply deformed at the moment of buckling. The critical load was determined by applying projection of the intersection onto the load-displacement and load-strain curves. Any analytical equation was not used to determine a shear buckling load in this study.

The evaluation results for the experimental buckling load by the data curves in Figures 10–13 were very consistent. Table 4 lists the evaluated shear buckling loads from the four data sets. Since the values were close enough, arithmetic means were calculated and listed. The determined shear buckling load was 295 kN.

### 3.3. Finite Element Analysis

**Figure 14.**
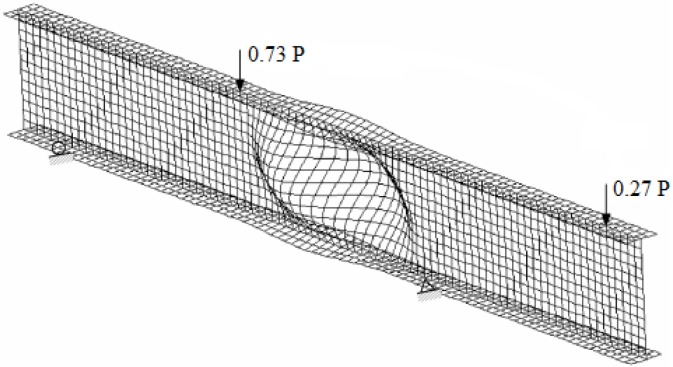
Finite element analysis; buckled shape of the deep composite I-shaped section.

The shear buckling load was also obtained using finite element computer program ABAQUS. The specimen was modeled three dimensionally using S8R5 element (8-node doubly curved thick shell, reduced integration). Figure 14 shows the finite element model of the I-shaped section. The specimen was modeled using a total of 1,800 elements and 6,345 nodes, which resulted in relatively fine mesh of which element aspect ratios were 1.08 for the web and 1.07 for the flange.

The orthotropic material properties determined using the coupon test results (Section 2.2) were used and the out-of-plane shear moduli (*G*_zx_ and *G*_yz_) were assumed to be the same as in-plane shear modulus (*G*_xy_). The loading and support points were identical to the experimental conditions. The bottom supports were modeled by restraining out-of-plane displacements and vertical movements. The node in the center of the bottom flange at a supported region was restrained against longitudinal translation to prevent a rigid body motion. The lateral (out-of-plane) displacements on the loading line were inhibited by the lateral supports and the loading areas on the top flange were also restrained in out-of-plane direction. The laterally supported areas on the web were also restrained in rotations and the displacement in the out-of-plane direction.

The total load *P* was divided into two forces *P*_1_ and *P*_2_ that had magnitudes of 0.73 *P* and 0.27 *P*, respectively to simulate the actual experimental loading conditions. The loads *P*_1_ and *P*_2_ were applied to the top flange as pressures on the area of 152 mm × 191 mm. This area was identical to that of the bearing plates placed on the top flange for the test. The stiffeners shown in Figure 4 were not included in the FEM modeling. Simplified displacement constraint conditions were applied instead: The lateral displacements of the web were assumed to be zero on the contact surfaces. The rotations about the longitudinal axis at the contacts were also fixed. Since the pure shear spot theoretically existed at the center of the test-section, the effect to the shear buckling load due to the usage of the stiffeners was not considered significant.

The shear buckling load was determined by taking the lowest positive eigen-value (first mode of buckling). The critical buckling load obtained from the finite element analysis was 292 kN. Figure 14 shows the buckled shape of the I-shaped section. The displacement magnification factor in the figure is 7.0. Additional post-processing figures with static and buckling analyses are also available [21].

## 4. Conclusions

In this study, the buckling of a pultruded polymer composite I-shaped section under shear loading was investigated experimentally. Based on the results, the following conclusions were derived:

1. The presented shear buckling test method using an asymmetric loading scheme effectively generated a shear loading condition and a pure shear region on the web of the tested I-section.
2. The corresponding displacements and shear strains were successfully obtained from the displacement and strain sensors, and they were used to observe the shear buckling behaviors of tested composite I-section.
3. In order to determine shear buckling load experimentally, a classical approach presented by Hoff, *et al.* was used. The results of shear buckling loads from load-lateral displacements relations, and load-shear strain at three different locations were very close.
4. The critical shear buckling load experimentally obtained was 295 kN.
5. In order to verify the experimental results, a finite element analysis was performed. The obtained shear buckling load was 292 kN. The analysis results support the validity of the presented experimental approach.

## Figures and Tables

**Table 1.** Average weight and volume fractions of the constituents (four samples each).

**Location**	**Constituents**	**Weight Fraction (%)**	STD ^*^ (%)	COV ^**^ (%)	**Volume Fraction (%)**	**STD (%)**	**CO (%)**
Web	Vinylester resin	43.4	1.8	4.1	56.2	1.6	2.8
	E-glass roving	24.3	1.3	5.3	16.7	1.1	6.6
	E-glass CSM	22.9	1.1	4.8	14.8	0.6	4.1
	Clay fillers	9.4	1.8	19.1	6.9	1.3	18.8
	Voids				5.4	0.7	13.0

Flange	Vinylester resin	46.2	0.7	1.5	57.4	0.6	1.0
	E-glass roving	7.8	1.2	14.7	4.8	0.8	15.6
	Carbon roving	14.0	1.2	8.4	12.6	1.0	7.7
	E-glass CSM	20.1	1.1	5.4	12.3	0.56	4.6
	Clay fillers	11.9	1.4	11.9	7.2	0.9	12.9
	Voids				5.7	1.0	16.7

* STD: Standard deviation;

** COV: Coefficient of variation.

**Table 2.** Mechanical property values of the flanges.

**Test**	**Loading direction**	**Constants**	**Average**	**COV (%)**
Flexural	Group I* (10 samples)	$E_{x}^{b}$	25.8 GPa	7.1
		$\sigma_{x}^{ub}$	347 MPa	5.5
		$\varepsilon_{x}^{ub}$	1.50%	11.5
	Group II^**^ (10 samples)	$E_{x}^{b}$	27.3 GPa	7.1
		$\sigma_{x}^{ub}$	436 MPa	5.3
		$\varepsilon_{x}^{ub}$	1.48%	16.2
Shear	*xy*-shear direction (10 samples)	*G_xy_*	3.8 GPa	6.4
		$\tau_{xy}^{u}$	99.5 MPa	3.4
		$\gamma_{xy}^{u}$	4.14%	10.2

* Group I
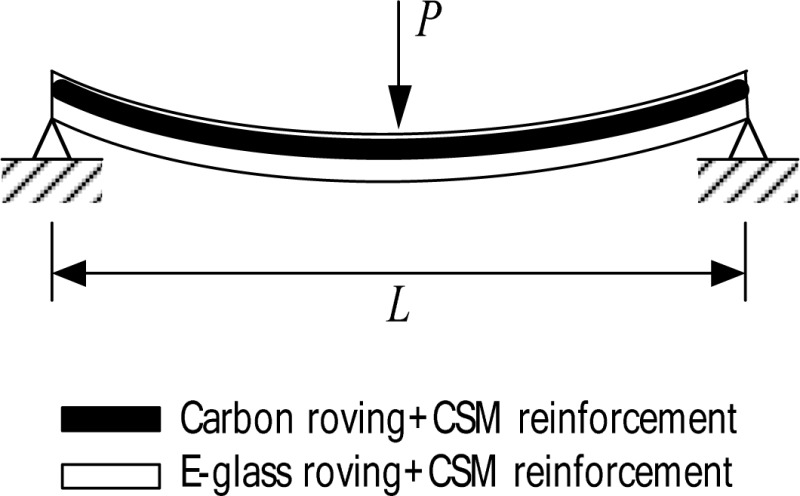

** Group II
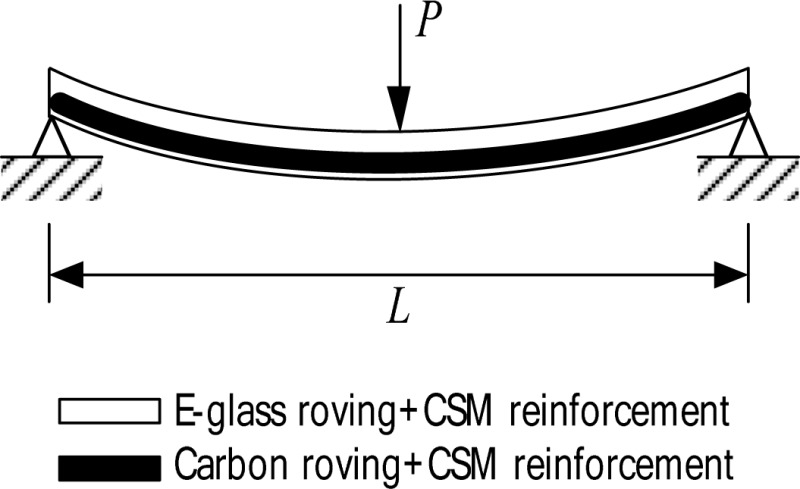

**Table 3.** Mechanical property values of the web.

**Test**	**Loading direction**	**Constants**	**Average**	**COV (%)**
Tensile	*x*-direction (10 samples)	$E_{x}^{t}$	18.9 GPa	6.9
		$\sigma_{x}^{ut}$	254.9 MPa	13.4
		$\varepsilon_{x}^{ut}$	1.44	5.1
		$\nu_{xy}^{t}$	0.29	3.9
	*y*-direction (10 samples)	$E_{y}^{t}$	9.99 GPa	2.3
		$\sigma_{y}^{ut}$	89.0 MPa	3.0
		$\varepsilon_{y}^{ut}$	1.44	7.8
		$\nu_{yx}^{t}$	0.18	10.0
Compressive	*x*-direction (10 samples)	$E_{x}^{c}$	20.4 GPa	7.4
		$\sigma_{x}^{uc}$	359.6 MPa	9.8
		$\varepsilon_{x}^{uc}$	1.65	6.3
		$\nu_{xy}^{c}$	0.33	6.7
	*y*-direction (10 samples)	$E_{y}^{c}$	11.5 GPa	6.7
		$\sigma_{y}^{uc}$	172.0 MPa	7.4
		$\varepsilon_{y}^{uc}$	1.88	22.9
		$\nu_{yx}^{c}$	0.20	5.5
Shear	*xy*-shear direction (10 samples)	*G_xy_*	4.3 GPa	9.3
		$\tau_{xy}^{u}$	96.9 MPa	6.2
		$\gamma_{xy}^{u}$	4.03	9.0

**Table 4.** The determined shear buckling loads (unit: kN).

	**Lateral displacements**			**Back to back Shear Strains at A-4**		**Back to back Shear Strains at B-2**		**Back to back Shear Strains at C-1**	
Sensor positions	W_1_	W_2_	W_3_	(γ*_xy_*)_1_	(γ*_xy_*)_2_	(γ*_xy_*)_1_	(γ*_xy_*)_2_	(γ*_xy_*)_1_	(γ*_xy_*)_2_
Shear buckling load	299	293	292	294	294	293	295	298	297
